# Stannous fluoride protects gingival keratinocytes against infection and oxidative stress by *Porphyromonas gingivalis* outer membrane vesicles

**DOI:** 10.3389/fdmed.2024.1492369

**Published:** 2024-11-19

**Authors:** Sancai Xie, Cheryl S. Tansky, Julie Ashe, Fei Gao, Nivedita B. Ramji, Vighter Iberi, Yiping Sun, Niranjan Ramji, Aaron R. Biesbrock

**Affiliations:** ^1^ Discovery & Innovation Platforms, The Procter & Gamble Company, Mason, OH, United States; ^2^ Global Oral Care R&D, The Procter & Gamble Company, Mason, OH, United States

**Keywords:** stannous fluoride, antibacterial agents, reactive oxygen species, *Porphyromonas gingivalis*, keratinocyte infection, periodontal diseases, scanning electron microscopy, transmission electron microscopy

## Abstract

**Objective:**

To determine whether outer membrane vesicles (OMVs) of the periodontal pathogen *Porphyromonas gingivalis* (*P. gingivalis*) can infect gingival keratinocytes and stimulate reactive oxygen species (ROS) production, and to assess whether stannous fluoride (SnF_2_), stannous chloride (SnCl_2_) or 0.454% SnF_2_ toothpaste diluents can inhibit OMV infection.

**Methods:**

OMVs were isolated from *P. gingivalis* culture and their morphology was characterized using scanning electron microscopy and transmission electron microscopy. OMVs were harvested, separated from parent bacteria, labeled with fluorescent probes, and added to proliferating gingival keratinocytes. Infection was monitored by measuring uptake of fluorescence. Free radicals and ROS were quantified by adding a separate CellROX fluorescent probe following 24 h incubation with OMVs, and automated fluorescence imaging was used to assess ROS generation rates. A dose response range of SnF_2_ and SnCl_2_ concentrations as well as 0.454% SnF_2_ toothpaste dilutions were added to OMVs to examine their potential to neutralize OMV infectivity and protect gingival keratinocytes from development of oxidative stress. The mechanism of SnF_2_ inhibition of OMV infection was studied by binding SnF_2_ with purified lipopolysaccharides (LPS) from the bacterial culture and examining the binding of stannous to LPS using mass spectrometry.

**Results:**

Large numbers of OMVs were formed in *P. gingivalis* culture medium. They were purified along with isolating soluble LPS. Fluorescence imaging revealed that OMVs infected gingival keratinocytes and promoted oxidative stress in a dose-dependent manner. SnF_2_, SnCl_2_, and SnF_2_ toothpaste inhibited OMV infectivity (*p* < 0.05) and likewise protected gingival keratinocytes from oxidative stress (*p* < 0.05). Stannous precipitated LPS and OMVs from solution, forming insoluble aggregates easily isolated by centrifugation. Mass spectroscopic analysis revealed that stannous was bound to LPS in a one-to-one molecular equivalent ratio.

**Conclusion:**

SnF_2_ not only kills bacteria, but also inhibits bacterial virulence factors, such as LPS and OMVs. SnF_2_, SnCl_2_ and stannous-containing toothpastes can precipitate OMVs and LPS to in principle protect gingival keratinocyte cells from infection leading to inflammation and oxidative stress.

## 1 Introduction

Periodontal diseases are epidemic worldwide. It is estimated that up to 50% of the global population exhibits some form of periodontal disease, which is elevated in older populations (1, 2). Gingivitis, the earliest stage of periodontal disease, presents with symptoms including swelling, redness, and bleeding of the tissues along the gingival margin (3, 4). In more advanced stages, the destruction of alveolar bone and ligament structures of the teeth result in tooth mobility and, in the worst cases, tooth loss (5, 6).

Periodontal diseases are the result of microbial dysbiosis in bacterial plaque biofilms that form above the gumline and in the gingival sulcus of developing periodontal pockets (3–6). The microbial biofilms in these locations prompt inflammatory responses in the gingival tissues, and it is the effects of the pronounced chronic inflammation and compromised host response that produce the tissue damage associated with periodontal disease. The dental plaque bacteria contributing to periodontal disease include a mixture of microorganisms with anaerobic species predominating as the disease progresses (7, 8). Microbes that appear dominant in the advancement of disease include the so-called “red three” gram negative anaerobic species; *Tannerella forsythia, Treponema denticola* and *Porphyromonas gingivalis (P. gingivalis)* (9, 10). *P. gingivalis* is a particularly important pathogen associated with the progression of periodontal disease (10–12). The local destruction of periodontal tissues caused by infection with this organism promotes increases in the flow of gingival crevicular fluid-containing serum (including heme) compounds and collagen-degradation products into the periodontal pockets. These changes in environmental conditions favor further growth of a mixture of pathogens in the subgingival microflora producing microbial dysbiosis (7, 12–15).

One of the virulence factors generally associated with gram negative anaerobic bacteria is their production of outer membrane vesicles (OMVs) (16, 17). OMVs are double layered spherical membrane-like bodies with diameters much smaller than the native bacteria, ranging in size from 50 to 250 nanometers (18). OMVs are continuously released from gram negative bacteria during their growth and proliferation (16, 19–22). In 1985, researchers first reported that *P. gingivalis* can produce OMVs, although at that time their physiological actions and pathogenic effects were not completely appreciated (23). The number of OMVs relative to parent *P. gingivalis* bacteria can be extreme. For example, the ratio of *P. gingivalis* bacteria to OMVs can range up to 1–2,000, and OMVs are adherent compared to their parent bacterium (24). Bacterial OMVs are comprised of lipopolysaccharides (LPS), phospholipids, outer membrane proteins and a portion of the periplasm that is captured during the membrane formation process (16–18). OMVs may form by a variety of mechanisms (16, 17, 25); however, regardless of their origin many components of OMVs are pathogenic and include factors which contribute to host cell immune system activation, destruction, immune system escape and host cell invasion (26). For example, OMVs present surfaces containing antigens to host tissues, most notably including endotoxin (e.g., LPS) (26). It has been suggested that virulence factors present in OMVs have advantages protecting them from host deactivation. Haurat et al. showed that *P. gingivalis* selectively package certain membrane proteins including gingipains into OMVs (27). Gingipains contribute to barrier function degradation of gingival tissues (28) and data show that gingipain levels in OMVs of *P. gingivalis* are 3–5 times those in the parent bacteria (29). Contemporary research has progressed to suggest that *P. gingivalis* OMVs play significant roles in the pathogenesis of periodontal diseases promoted by this bacteria (26).

Our laboratories have been carrying out research to understand the magnitude, duration and mechanisms of the high bioavailable stannous fluoride (SnF_2_) formulated in dentifrices for the prevention of periodontal diseases. In a previous study, we evaluated the histomorphology of SnF_2_ reactivity with *P. gingivalis* bacteria using TEM and identified significant deposition of insoluble aggregates in cell membranes leading to lysis and bactericidal activity (30). We posited that the reactivity of SnF_2_ with *P. gingivalis* was most likely associated with precipitation of endotoxin and phospholipids in cell membranes (30). Notably, LPS reactivity with SnF_2_ has been shown to reduce the antigenicity of reacted LPS to toll receptor promotion of inflammation pathways (31–33). During the aforementioned TEM study (30) we observed significant numbers of OMVs in incubation mixtures of *P. gingivalis* and we likewise saw significant SnF_2_ reactivity with membranes of these vesicles. The purpose of this study was to expand upon those learnings and carry out a more comprehensive study, specifically to establish if OMVs of *P. gingivalis* can infect and stimulate reactive oxygen species (ROS) production in gingival keratinocytes and to assess whether SnF_2_, stannous chloride (SnCl_2_) or 0.454% SnF_2_ toothpaste diluents can inhibit OMVs. TEM analyses were performed to assess the location and tenacity of SnF_2_ interactions with *P. gingivalis* matrix vesicles. In addition, OMVs treated with SnF_2_ and SnCl_2_ were examined to see if reactivity diminished the inflammatory promotion of OMVs with gingival keratinocytes using ROS generation as a probe for inflammation promotion. Lastly, mass spectrometry analysis was carried out to confirm the nature of SnF_2_ reactivity with LPS.

## 2 Materials and methods

### 2.1 Bacterial growth and OMV isolation

*P. gingivalis* (ATCC catalog #33277, American Type Culture Collection, Manassas, VA) was cultured in 30 ml MTGE media (Anaerobic Enrichment Broth, Anaerobe Systems, 6 ml tubes- catalog #AS-778 & 500 ml bottles- catalog #AS-7785, Anaerobe System, Morgan Hill, CA) in a sterile 125 ml Erlenmeyer flask under anaerobic conditions at 37°C for 48 h as seeding bacteria. The seeding bacterial culture was inoculated with seven liters of fresh MTGE media and continued to grow for 48 h under anaerobic conditions at 37°C. The bacteria were harvested at the end of culture by centrifugation in a JA-10 rotor at 10,000 g, 4°C for 60 min in Avanti J-26 XPI High-Performance Centrifuge of Beckman Coulter (Indianapolis, IN). The supernatant was collected and filtered through 0.45 μm pore PVDF membranes to remove cell debris.

OMVs were secreted by *P. gingivalis* into the MTGE media. To isolate OMVs, the conditioned culture medium volume was reduced by filtration using a tangential flow filtration Minimate TFF System (PALL Life Sciences, Port Washington, NY) with filter capsules of molecular weight cutoff 100 kD at 40 Psi. The retentate of the filtration was centrifuged at 140,000 × g for 1 h at 4°C using an SW32 swinging bucket rotor on a Beckman XL-100 K Ultracentrifuge (Beckman Coulter, Atlanta, GA) to separate the OMV pellet from the supernatant. The supernatant was retained as the starting material for secreted LPS purification (see below). The pellets were resuspended in dPBS buffer [1X Dulbecco's Phosphate Buffered Saline (dPBS): Life Technologies, Grand Island, NY] and centrifuged at 200,000 × g for 1 h at 4°C (using an SW41 swinging bucket rotor) to yield a standard OMV preparation.

To generate highly pure OMVs, the initial OMVs from the first ultracentrifugation were then resuspended in 800 μl HEPES buffer (50 mM HEPES, 150 mM NaCl, pH 6.8, Life Technologies, Grand Island, NY), stained with Vybrant™ Multicolor Cell-Labeling Kit with DiD Solutions (ThermoFisher Scientific, Waltham, MA, USA), and underwent another round of ultracentrifugation using OptiPrep™ (60% w/v iodixanol in water, Sigma-Aldrich, St. Louis, MO, USA) discontinuous density gradient. The initial OMV preparation was separated into four samples and each resuspended in 3 ml HEPES buffer containing 45% w/v iodixanol and placed in 4 Ultra-Clear™, 14 ml, 14 × 95 mm tubes (Beckman Coulter, Atlanta, GA). A discontinuous iodixanol gradient was achieved in each sample by layering successive 1.5 ml of HEPES buffer containing 45%, then 40%, 35%, 30%, 25% & 20% w/v iodixanol, with 45% at the bottom, in a total of 9.5 ml. Tubes were centrifuged at 173,000 × g for 72 h at 4°C using a 70.1Ti rotor installed in a Beckman XL-100 K Ultracentrifuge (Beckman-Coulter, Atlanta, GA). Eight 0.5 ml gradient fractions from each sample (1, 2, 3 & 4) were collected from top to bottom of the density gradient solution and measured at A260 and A280 for DNA/RNA and proteins, respectively, using an 8-channel NanoDrop spectrophotometer according to the manufacturer's instructions (ThermoFisher Scientific). OMVs containing fractions were identified by measuring endotoxin contents using the Pierce™ LAL Chromogenic Endotoxin Quantitation Kit, per manufacturer's instructions (ThermoFisher Scientific).

Fractions containing the purified OMVs were washed with endotoxin-free water (Sigma, St. Louis, MO) and centrifuged twice at 200,000 × g for 2 h at 4°C using a SW40 Ti rotor installed in a Beckman XL-100 K Ultracentrifuge (Beckman-Coulter, Atlanta, GA). The highly pure OMVs were resuspended in 30 ml of endotoxin-free water, and aliquoted into 0.5 ml Eppendorf tubes and stored at −80°C. OMVs were quantified using Bradford assay to estimate the amounts of proteins. OMVs were subsequently fluorescently labeled (see below) to assess their integration and infection into gingival keratinocytes.

### 2.2 Fluorescence-labeling of OMVs

OMVs were labelled with either green (Lipophilic Tracers DiO) or red (Lipophilic Tracers DiD) fluorescence dye following the manufacturer's instructions (Invitrogen™ Vybrant™ Multicolor Cell-Labeling Kit with DiO, DiI, DiD Solutions, 1 ml each, ThermoFisher Scientific). Briefly, the lipophilic dyes were added directly to the OMVs. The mix was incubated at 37°C for 20 min under a light proof condition. Unlabeled dyes were removed using Sephadex LH-20 resin in a Spin Columns-Snap Cap with a Collection Tube (ThermoFisher Scientific).

### 2.3 SnF_2_ and SnCl_2_ solution and SnF_2_ toothpaste supernatant reactivity

For compound treatment, SnF_2_ and/or SnCl_2_ (Sigma, St. Louis, MO) were freshly weighed and dissolved in UPW water at 10 mM as stock solutions on the day of treatment. They were then added to cell culture medium. For toothpaste diluents treatments, SnF_2_ 0.454% W/W (Crest Pro-Health Advanced Gum Restore Deep Clean Toothpaste and Crest Pro-Health Enamel Repair and Gum Toothpaste, Procter & Gamble, Cincinnati, OH) were used to prepare diluted slurries with pure water. Toothpaste solution was freshly prepared right before the experiment in anaerobic conditions. Toothpaste was weighed and dissolved in UPW water at 10% as a stock solution. Vigorous vortexing of toothpaste stock was done for 30 min in flasks to assure complete suspension. Then 1 ml of the toothpaste suspension was transferred to a microtube and centrifuged at high speed (12,000 g) for 10 min at 4°C. The supernatant was collected into a 15 ml tube and diluted with complete cell culture medium for treatment.

### 2.4 Gingival keratinocyte cell culture and treatment

Immortalized Human Gingival Keratinocytes were purchased from Applied Biological Materials Inc. (Richmond, BC, V6V 2J5, Canada). Cells were maintained in a complete growth medium (proprietary to Applied Biological Materials Inc) under an atmosphere of 5% CO_2_ and 95% air, 95% humidity at 37°C. For OMV infections, cells were harvested from flasks at 60%–80% confluence, counted using a cell counter (Invitrogen™ Countess™ 3 Automated Cell Counter, ThermoFisher Scientific) and suspended in fresh complete growth medium. The cells were seeded at 7,000 in 100 μl of culture medium in each well of a 96-well plate. Fluorescence-labelled OMVs were subsequently added to the cell culture the next day. Both the stannous solutions and the toothpaste supernatants were diluted with complete cell culture medium and mixed with OMVs. These mixtures were incubated for 10 min at room temperature before application. The cells were then fed with the 100 μl of the mixture, incubated in the Incycyte S3 (Sartorius, Bohemia, New York), which was placed inside a regular incubator with an atmosphere of 5% CO_2_ and 95% air, 95% humidity at 37°C.

### 2.5 OMV infection of gingival keratinocytes. ROS generation following OMV infection of gingival keratinocytes

OMVs bind Toll-like receptors on the surface of, and inside, the gingival keratinocytes and modulate their metabolic pathways. Consequently, free radicals and oxidative species are generated within the cells. The internalization of OMVs into keratinocytes was recorded by taking images at preset intervals. To quantify the oxidative molecules within the cells, we applied CellROX fluorescence dye to the cells 24 h after addition of the OMVs to the cells. Both Deep Red and Green CellROX dyes (ThermoFisher Scientific) are fluorogenic probes for quantifying ROS in live cells. CellROX Green Reagent is weakly fluorescent while in a reduced state and exhibits bright green photostable fluorescence upon oxidation by ROS and survives detergent treatment. CellROX Deep Red Reagent is also a cell-permeant dye. It is non-fluorescent in a reduced state, and exhibits bright fluorescence upon oxidation by ROS. To make sure that SnF_2_, SnCl_2_ and toothpaste supernatants did not quench specific dyes, we used both Deep Red and Green CellROX reagents in our experiments. CellROX dyes (2.5 μM) were added to cells 24 h after addition of OMVs into cell culture. Images were taken at a regular interval for fluorescence quantification.

### 2.6 Imaging

Green and red fluorescence and phase contrast images were acquired every one to three hours for the first 24 h, every hour after adding the CellROX dyes for 10 h, and then every two hours for 3 days with an objective of 10X or 20X in the Incycyte S following the manufacturer's instruction. Three images were taken in each well at pre-selected sites in a 96-well plate, where cells were likely evenly distributed. Fluorescence-labeled OMVs were imaged in the focus plane of the objective. The CellROX™ Green or Red Detection Reagent is cell-permeable and non-fluorescent or very weakly fluorescent while in the reduced state. The fluorescent dyes bind DNA and generate strong fluorogenic signals. The images were taken and analyzed using the software from the manufacturer. Two dyes, Vybrant™ DiO Cell-Labeling and Vybrant™ DiD Cell-Labeling dyes, were used to label the OMV. Two ROS detection dyes, CellROX™ Green and CellROX™ Red, were used to visualize the oxidation conditions inside gingival keratinocytes. The fluorescence signals were different between the four fluorescence dyes. For comparisons, the fluorescence signals were normalized by dividing raw fluorescence counts with the mean fluorescence counts of 0.65 OMV μg/ml at 36 h in each plate. All images of CellROX oxidation were exported from instrument Incycyte S at 36 h. The time stamps (0 d 0 h 0 m) were default when exporting individual images.

### 2.7 Isolation of secreted LPS from *P. gingivalis*

The supernatant collected from OMV isolation was used as the source of secreted LPS from the bacterium. Secreted LPS were extracted and purified using previously described procedures (34, 35). Briefly, bacteria and bacterial fragments were removed using centrifugation and consecutive filtration with 0.45 μm and 0.22 μm membrane filters. OMVs were then concentrated from 7 liters to 100 ml using tangential flow filtration with filter capsules of a 100 kD molecular weight cutoff at 40 Psi. The concentrated OMV preparation was diluted with 500 ml of water and reconcentrated using tangential flow filtration to remove any molecules smaller than 100 kD. Individual LPS molecules, which are around 10–20 kD, would be filtered out, while only LPS aggregated as vesicles were retained. This dilution process was repeated twice to ensure that only bacterial components larger than 100 kD remained in the OMV preparation. The concentrated OMV preparation was then pelleted by ultracentrifugation, and the supernatant was saved for LPS isolation. It is likely that LPS in the supernatant existed in some form of vesicles that were not precipitated during ultracentrifugation. The supernatant was adjusted to 300 ml buffer containing 10 mM Tris-Cl buffer (pH 8), 2% Sodium Dodecyl Sulphate, 2 mM MgCl_2_ and 40 mg Proteinase K (all chemicals and proteinase K were purchase from Sigma, St. Louis MO). The mixture was vortexed and placed an incubator at 68°C for 24 h. Sixty ml of 3 M sodium acetate pH 5.2 and 800 ml 100% ethanol were added and kept at −20°C. The crude LPS preparation was precipitated using a JA-10 rotor at 13,000 RPM, 4°C for 60 min in Avanti J-26 XPI High-Performance Centrifuge of Beckman, and was further purified using affinity chromatography following procedures described by Hirayama et al. (36) and Sakata et al. (37) The endotoxin activities were analyzed in the fractions using the Pierce™ LAL Chromogenic Endotoxin Quantitation Kit, per manufacturer's instructions (ThermoFisher Scientific).

### 2.8 OMV and SnF_2_ precipitation assay

*P. gingivalis* 33,277 OMV was used for the precipitation assay. SnF_2_ solution was freshly prepared in each experiment in water. OMV, prepared in water, was added to the wells of a 96-well plate first, and then SnF_2_ was added. The plate was transferred to spectrometry reader (Molecular Devices SpectraMax M5 Multi-Mode Microplate Reader, Molecular Devices, LLC, San Jose, CA) for OD600 kinetic reading in 10 min intervals.

### 2.9 Mass spectrometry—LPS binding measures

Matrix-assisted laser desorption ionization (MALDI) is a soft ionization technique used in mass spectrometry (MS) (38). MALDI mass spectrometry can be used for the analysis of biomolecules, including peptides, proteins, polysaccharides, and large organic molecules and polymers. In MALDI, the analyte is first co-crystallized with a UV-absorbing matrix such as alpha-cyano-4-hydroxycinnamic acid, then subjected to pulse laser radiation. This causes the desorption of the analyte/matrix crystals and produces ions which are transmitted into a mass analyzer for detection. In MALDI TOF, a time-of-flight (TOF) mass analyzer is used. MALDI TOF data can be acquired in MS mode to generate molecular weight information and in MS/MS mode to generate structural information. Typically, MALDI mass spectrometry data acquisition takes less than a minute, so the technique can be used to quickly screen for molecular species in samples of interest.

In this study, *P. gingivalis* secreted LPS molecular weight profiles and their interaction with SnF_2_ were investigated via MALDI TOF. An equal volume of soluble LPS solutions with or without added SnF_2_ was mixed with alpha-cyano-4-hydroxycinnamic acid (10 mg/ml in 80% acetonitrile/20% water). Next, 0.7 ul of the sample solution was spotted on a MALDI plate, air-dried, and analyzed in negative-ion mode using a MALDI TOF/TOF 4,800 *Plus* Analyzer (AB-SCIEX, Framingham, MA, USA). Data were acquired using a mass scan range of 500–3,000 Da and a laser power of 4,500. Data were collected in an automated fashion using random sampling over the sample spot with 250 shots per subspectrum and a total of 2,500 shots per spectrum. The mass of LPS corresponded to part of the mass of Lipid A in the LPS molecules, as shown previously (40–42).

### 2.10 TEM sample preparation and imaging

*P. gingivalis* isolated OMVs were placed in a fixative solution (2% glutaraldehyde in PBS buffer) immediately, and stored at 4°C. The fixed samples were post-fixed with 1% OsO_4_ in PBS buffer overnight at 4°C. The samples were then hydrated by gradually increasing the concentration (50%, 70%, 85%, 90%, 100%) with ddH_2_O for 3 h at room temperature and filtrated by gradually increasing the concentrate of Epon 812 with acetone (30%, 50%, 75%, 100%) at room temperature. All samples were embedded in Epon 812 Epoxy (Electron Microscopy Sciences, Hatfield, PA) and cured overnight at 65°C. Each sample block was trimmed, sectioned by Leica ultramicrotome (Leica Microsystems, Deerfield, IL) a Diatome ultracut 45° diamond knife. Roughly 70 nm thick sections were collected, placed on 200 mesh copper grid with formvar, and post-stained with uranyl acetate for 30 min and lead citrate for 10 min. All samples were examined by Hitachi S5200 STEM (Hitachi High-Tech, Hillsboro, OR) for high resolution imaging analysis at 30 KV and with Bruker EDS detector (Bruker, Madison, WI) for elemental information.

### 2.11 SEM sample preparation and imaging

Bacterial cultures were centrifuged to collect the bacteria after 48 h, and the bacterial pellets were placed in a fixative solution (2% glutaraldehyde in PBS buffer) immediately and stored at 40°C. The fixed samples were post-fixed with 1% OsO4 in PBS buffer overnight at 40°C. The samples were then dehydrated by gradually increasing the concentration (50%, 70%, 85%, 90%, 100%) with ddH2O for 3 h at room temperature. The dehydrated samples were deposited onto a filter paper and secured with silver paint on a sample mount. Additional sputter-coating with Au/Pd was performed in a Gatan Alto 2,500 (Ametek, Berwyn, PA) for 60 s. High-resolution imaging was performed in a Hitachi S-4700 (Hitachi High-Tech, Hillsboro, OR) SEM with an accelerating voltage of 3 kV.

### 2.12 Statistical analysis

Results were organized and analyzed using R. Analysis of variance (ANOVA) was performed first to analyze the whole set of data. When significant variations were found, a *t*-test was used to assess the differences between groups using the stat_compare_means function in the ggpubr package for bar plots. For line plots, ANOVA was performed, and differences between treatment groups were compared using pairwise *t*-test, with *p*-values adjusted using the Bonferroni method. Two fluorescent dyes, Lipophilic Tracers DiO (green) and DiD (red), were used to stain the OMVs. Additionally, two CellROX dyes (green CellROX and red CellROX) were employed to monitor ROS contents in the cells, ensuring the validity of the fluorescence readings. For statistical analysis, fluorescence readings were normalized in each experiment as relative fluorescence. The fluorescence reading was normalized with 0.65 µg/ml OMV at 36 h, where the relative fluorescence of 0.65 µg/ml OMV at 36 h was set at 100.

## 3 Results

### 3.1 OMV isolation

**Figure 1:**
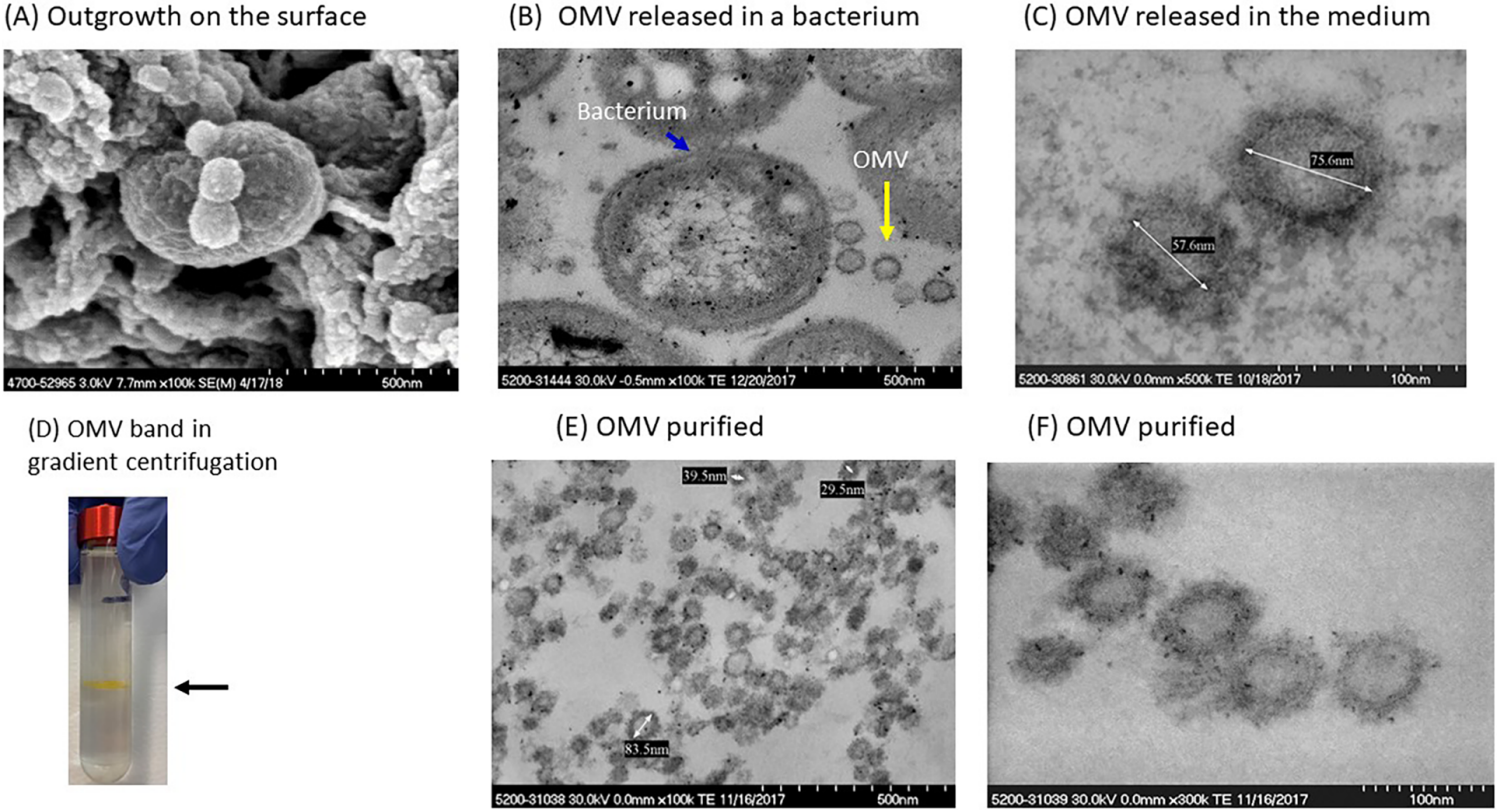
Isolation and characterization of *P. gingivalis* OMVs. **(A)** SEM of *P. gingivalis* bacteria with small outgrowths on surface. **(B)** Several OMVs (yellow arrow) adjacent to a *P. gingivalis* bacterium (blue arrow). **(C)** *P. gingivalis* OMVs in the culture medium. **(D)** A visible band of OMVs in OptiPrep gradient gel. **(E,F)** Purified OMVs of *P. gingivalis*.

Figure 1A shows an SEM of *P. gingivalis* capturing the formation of OMVs on the surface of the bacterium. TEM images in Figure 1B show ubiquitous OMVs being created in *P. gingivalis* culture. Dimensions of the vesicles are shown in Figure 1C. OMVs were isolated from growth cultures of *P. gingivalis* bacteria using gradient ultracentrifugation. A visual example of the separated band is shown in Figure 1D. The OMVs were then purified and resuspended in media as described in the Methods. Figures 1E,F show a collection of suspended OMVs as illustrated by the TEM microscopic image.

### 3.2 Entry of OMVs into gingival keratinocytes

**Figure 2:**
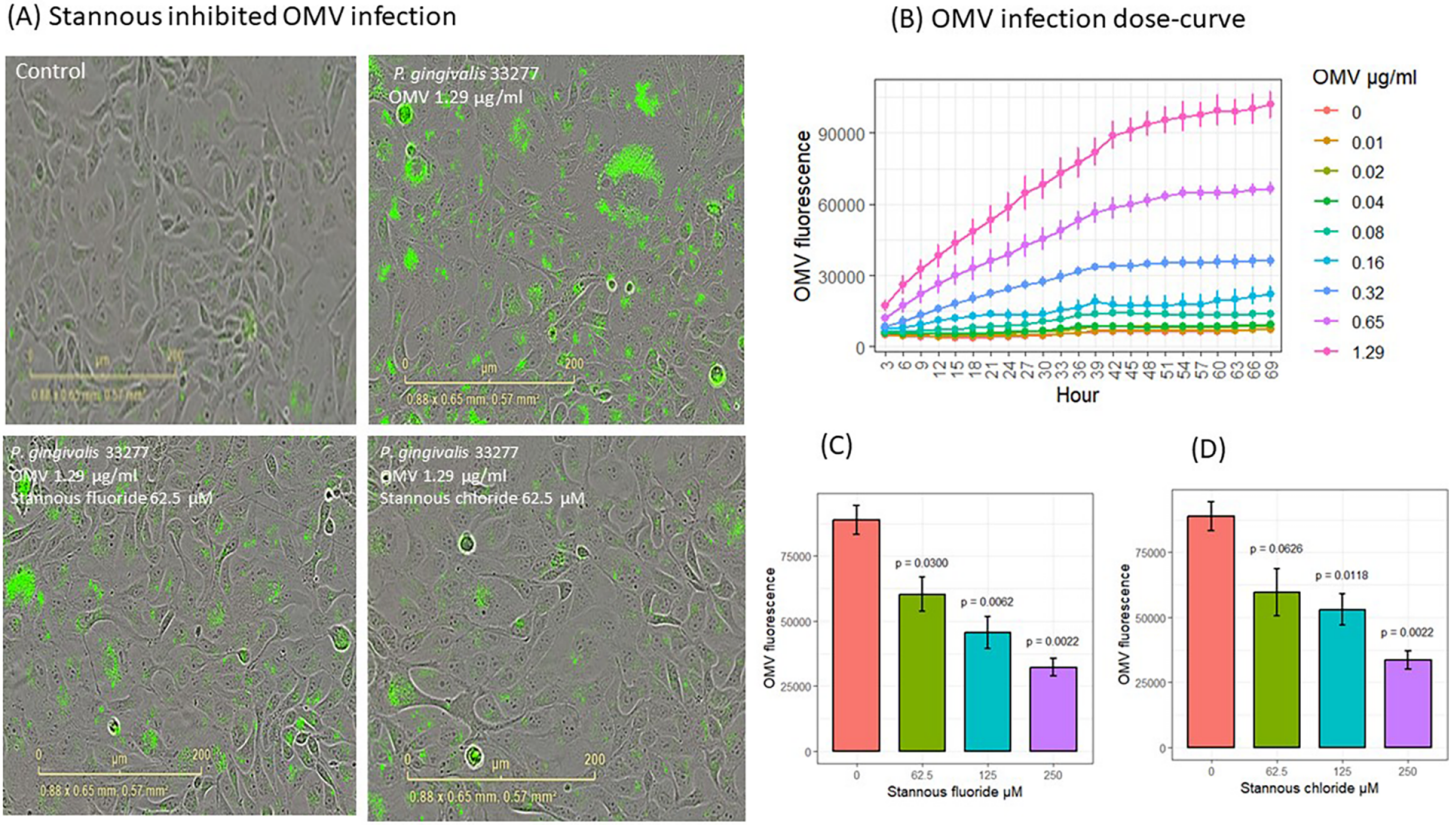
SnF_2_ and SnCl_2_ inhibited OMV entry into gingival keratinocytes. **(A)** Images of fluorescent-labeled OMVs (green) entry into gingival keratinocytes. **(B)** Entry of OMVs into gingival keratinocytes in a dose curve. Each time point represented the mean and SE of three separate experiments. **(C)** SnF_2_ and **(D)** SnCl_2_ inhibited entry of OMVs into gingival keratinocytes at 42 h. Each bar represented the mean and SE of three separate experiments. The plots were generated using ggpubr package in RStudio. The results were analyzed using one-way ANOVA and *t*-test conducted to compare the difference with OMV alone (stannous is 0 μM) as the reference group in stat_compare_means of Rstudio packages.

Fluorescence-stained OMVs were suspended in the culture medium. The microscopy objective captured images only in the focused plane, ensuring that only fluorescence-stained OMVs attached to or internalized by cells were visible (Figure 2A). OMVs entered gingival keratinocytes in a dose- and time-dependent manner (Figure 2B). Significant increases in OMV entry were observed (*p* < 0.05) at 3 h with 0.65 and 1.29 μg/ml OMVs, at 12 h with 0.32 μg/ml OMVs, and at 63 h with 0.16 μg/ml OMVs. OMVs did not have obvious deleterious effects in the first 69 h of OMV infection at the doses used in the dose curve study (Supplementary Figure S1, *p* < 0.05). It is worth noting that *P. gingivalis* OMVs are highly virulent and kill almost all gingival keratinocytes after about 100 h of infection through either pyroptosis or apoptosis (unpublished results from our labs) at concentrations of 0.75 μg/ml or higher in separate experiments.

To understand the effect of stannous on OMV internalization into keratinocytes, we analyzed the fluorescence results at 42 h. SnF_2_ inhibited the entry of OMVs at 1.29 μg/ml into gingival keratinocytes at all three concentrations tested (62.5, 125, and 250 μM, *p* ≤ 0.030; Figures 2A,C). Similarly, SnCl_2_ also reduced OMV internalization at 125 and 250 μM (*p* ≤ 0.012; Figure 2D).

We analyzed cell proliferation and growth using phase contrast images at 42 h. Neither SnF_2_ nor SnCl_2_ reduced cell numbers at 42 h, as shown in Supplementary Figure S1B.

### 3.3 Oxidative stress

**Figure 3:**
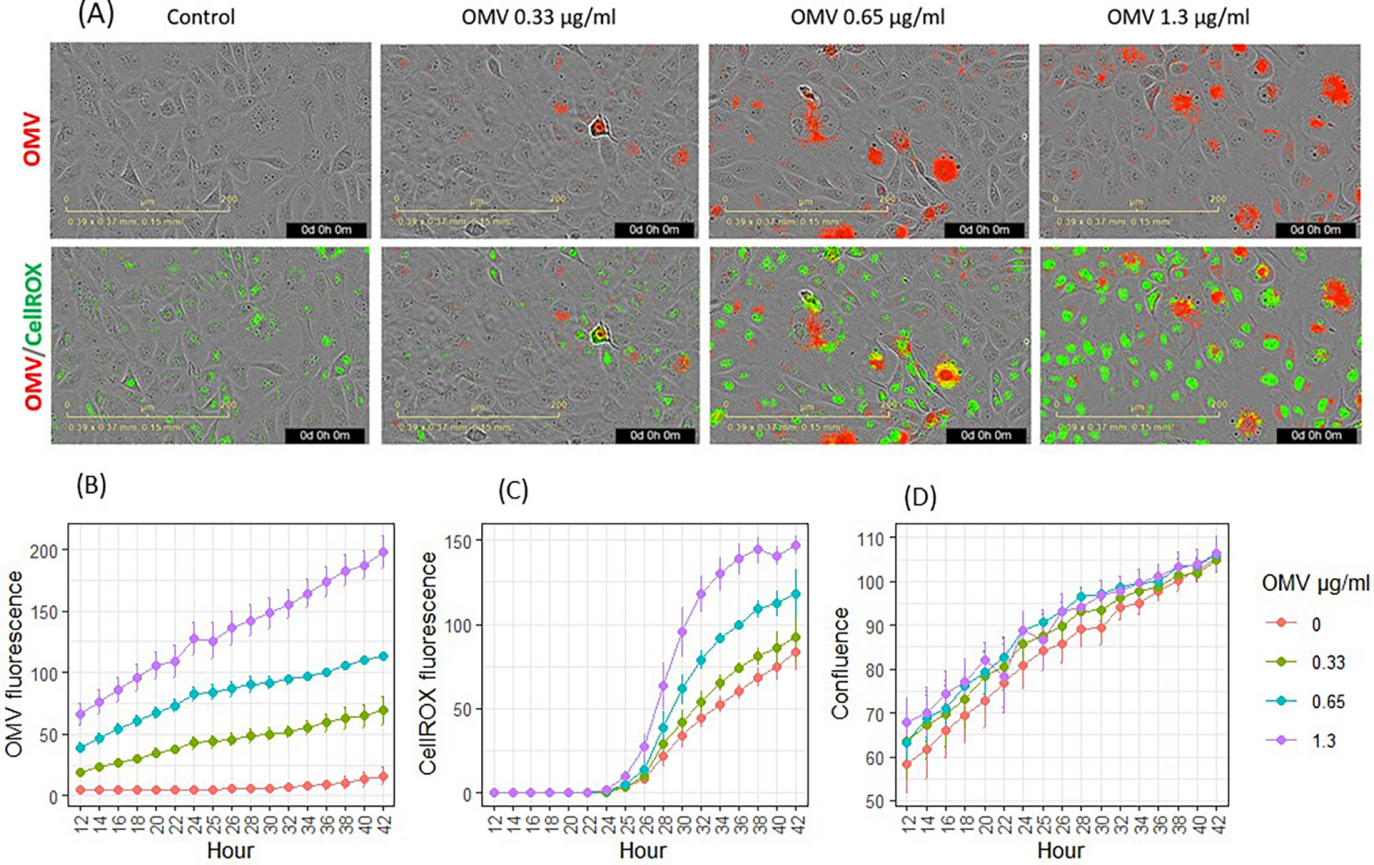
Effects of *P. gingivalis* OMVs on oxidation of CellROX dyes in gingival keratinocytes. **(A)** Images of OMV (red) infection and oxidation of CellROX (green). **(B)** Entry of OMVs into gingival keratinocytes. **(C)** Oxidation of CellROX dyes in gingival keratinocytes. **(D)** Cell confluence evaluated by phase contract images. Each time point represented the mean and SE of five separate experiments.

Following OMV exposure, keratinocytes exhibited signs of infection, as shown by images highlighting fluorescence dye-labeled OMVs (red in Figure 3A). OMVs modulated metabolic activities and increased ROS, such as hydroxyl radicals. CellROX dye was added to the cells after 24 h of incubation with OMVs. ROS oxidized the CellROX into fluorescent products (green in Figure 3A). The entry of OMVs into cells (Figures 3A,B) followed the same pattern as in the previous experiment (Figure 2B). OMV fluorescence increased inside the cells in a time- and dose-dependent manner (*p* < 0.05 for all concentrations from hours 14 to 42). OMVs increased CellROX fluorescence in a dose- and time-dependent manner (Figures 3A,C). OMVs increased cellular oxidation from 30 to 42 h for 1.3 μg/ml (*p* < 0.05) and from 32 to 40 h for 0.65 μg/ml (*p* < 0.05, Figure 3C).

Both fluorescence-labeled OMVs and CellROX stain did not significantly reduce cellular coverage, as evaluated using phase contrast imaging from hours 14 to 42.

**Figure 4:**
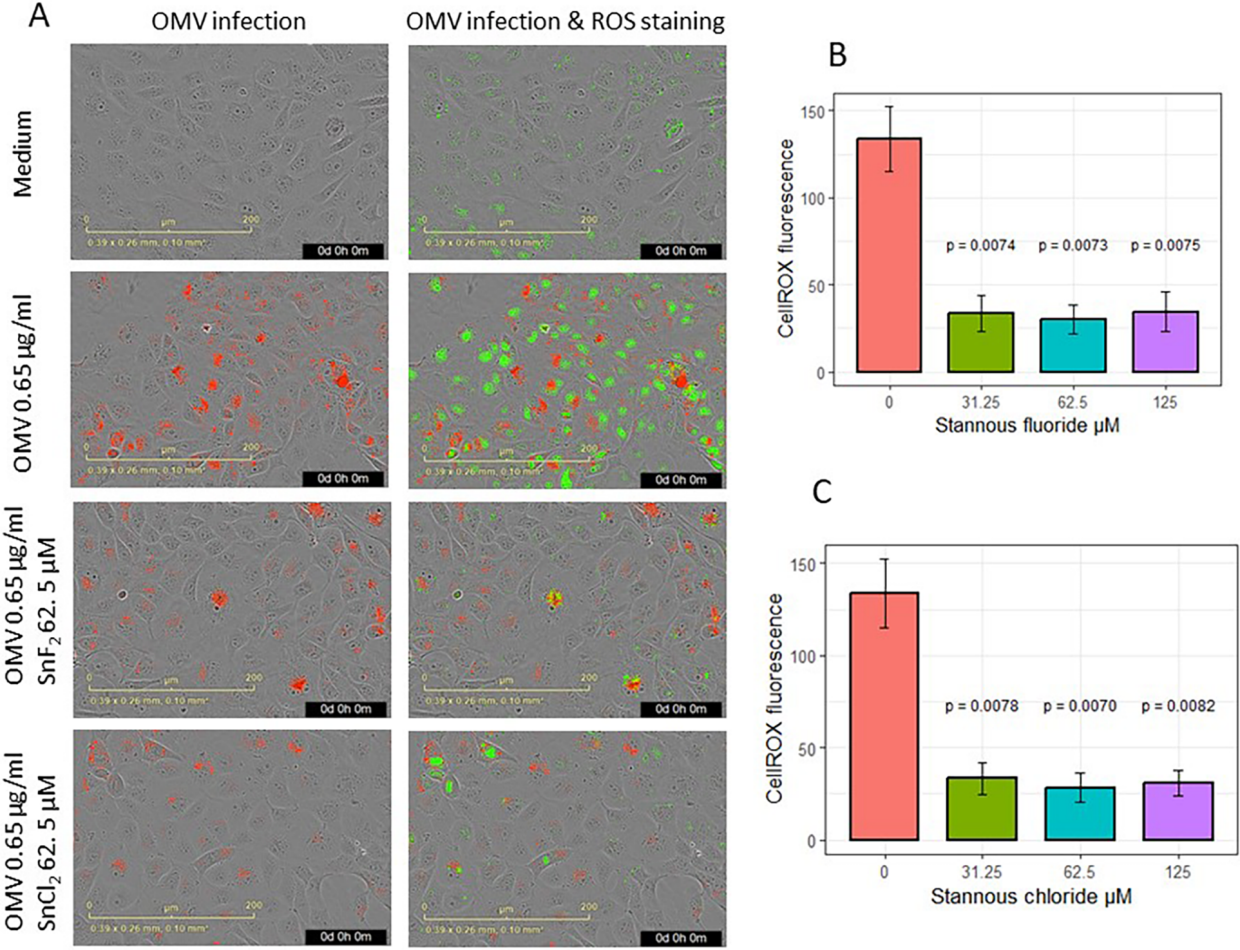
Effects of SnF_2_ and SnCl_2_ on the intake of fluorescence-labeled OMV (Red) and oxidized CellROX Dye (green): **(A)** Images of OMV intake and oxidized CellROX green in cells infected with 0.65 μg/ml OMVs without or with SnF_2_ or with SnCl_2_. **(B)** SnF_2_ and **(C)** SnCl_2_ inhibited cellular oxidation of CellROX. Each point represents the mean and SE from four separate experiments at 42 h. CellROX fluorescence was expressed as relative fluorescence. *P*-values listed here were from pairwise comparisons with the stannous 0 µM (OMV at 0.65 µg/ml alone) as the reference group.

The effects of SnF_2_ and SnCl_2_ on OMV infection of keratinocytes and the subsequent generation of ROS are shown in Figure 4. Both SnF_2_ and SnCl_2_ inhibited the oxidation of CellROX in gingival keratinocytes (Figures 4B-C; *p* ≤ 0.008 for all three concentrations tested). Additionally, SnF_2_ and SnCl_2_ did not significantly change cell numbers (Supplementary Figure S2A). SnF_2_ concentrations in media containing OMVs produced substantial reductions in ROS production. These effects were observed at SnF_2_ concentrations as low as 31.25 μM, well within the range observed for SnF_2_ penetration into the subgingival sulcus following brushing with SnF2 toothpaste (42).

**Figure 5:**
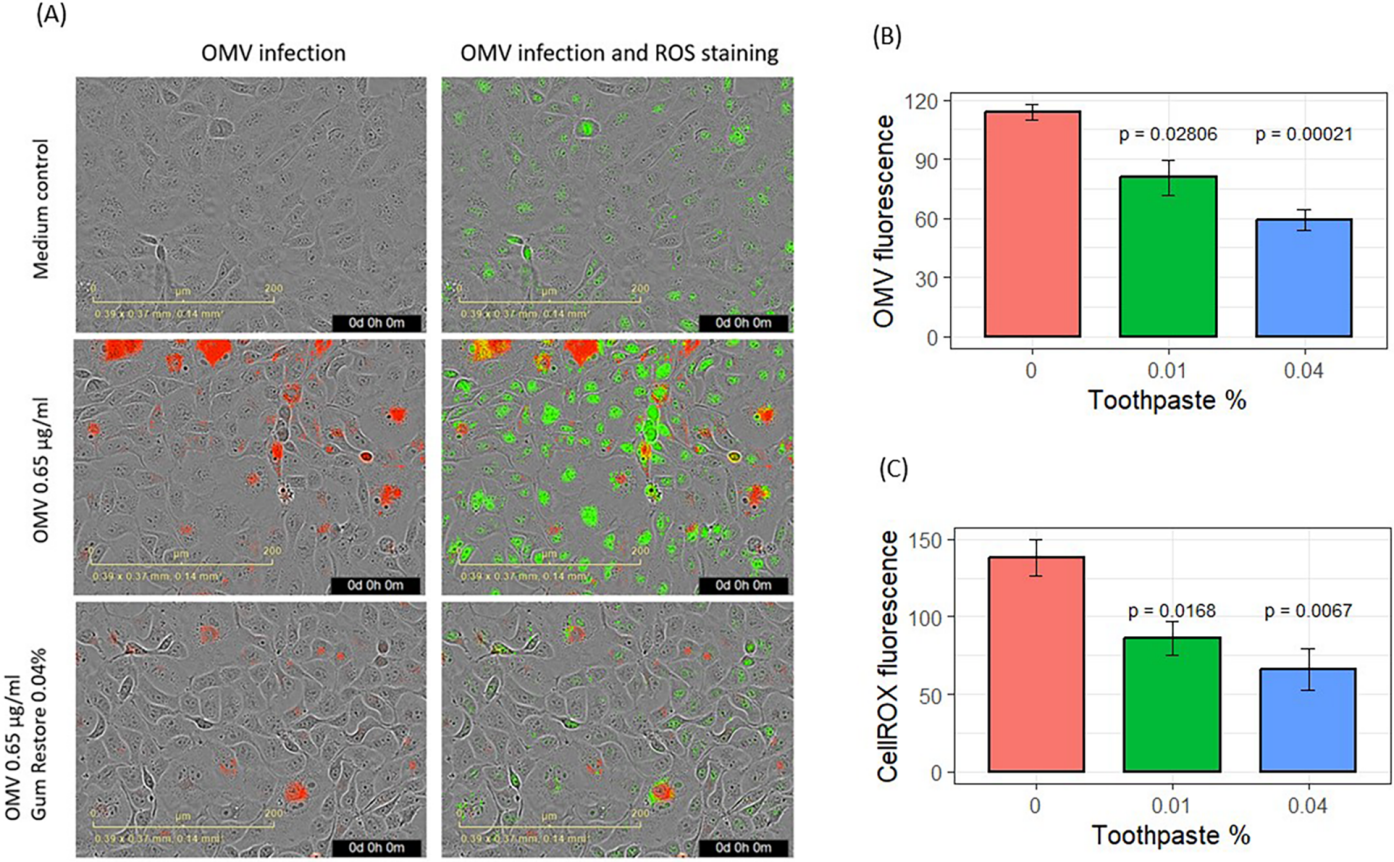
Effects of stannous-based toothpastes on OMV entry and oxidative stress. **(A)** Images showing OMV (red) entry into gingival keratinocytes and oxidized CellROX Green stains (green). **(B)** Entry of fluorescence-labeled OMVs into gingival keratinocytes. **(C)** Quantification of oxidized CellROX dyes inside the cells. CellROX fluorescence and OMV fluorescence are presented as relative fluorescence. Results from two SnF_2_-containing toothpastes were pooled for analysis. Each bar represents the mean and SE of four separate experiments.

Results for the effects of SnF_2_ toothpaste supernatants on OMV infection and inflammatory promotion are shown in Figure 5. The effects of the two toothpastes were similar, so the results were pooled as shown in Figure 5. Addition of dentifrice supernatant at 0.01% and 0.04% dilution produced significant reductions in OMV infectivity of keratinocytes (*p* ≤ 0.028) and suppressed subsequent generation of ROS (*p* ≤ 0.017), as shown in Figures 5A–C. Cell confluence was not significantly impacted (Supplementary Figure S2C) by addition of toothpaste supernatants, which is consistent with our unpublished results from other experiments.

**Figure 6:**
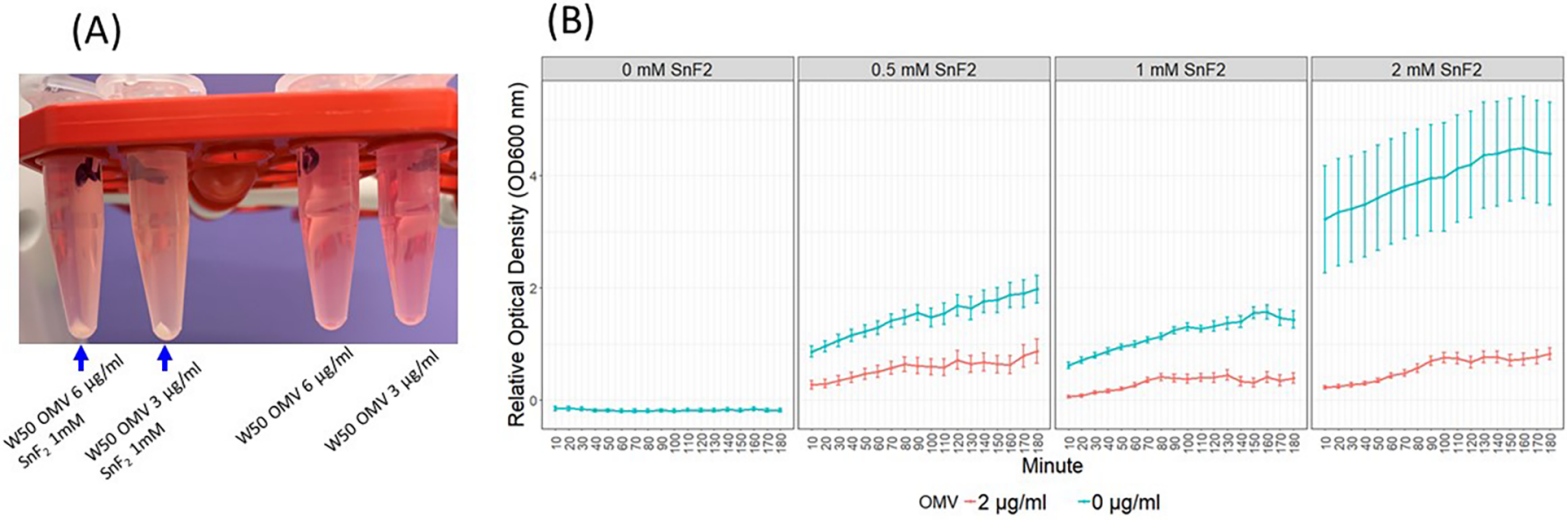
OMVs of *P. gingivalis* and SnF_2_ formed precipitates. **(A)** SnF_2_ binds OMVs and forms precipitates. **(B)** Time course of stannous binding to OMVs. Each time point represents the mean and SE of 24 independent measurements. 0 mM SnF_2_ was not represented in B since the OD600 was 0. Relative OD600 values were obtained by dividing OD600 by the average OD600 of the 2 µg/ml OMV + 1 mM SnF_2_ samples at the 60 min timepoint. Mixed effect models were fitted to the relative OD600, and all comparisons were significant at 0.01 after Bonferroni adjustment. These comparisons include SnF_2_ + OMV vs. SnF_2_, OMV + 0.5 mM SnF_2_ vs. OMV, and OMV + 1 mM SnF_2_ vs. OMV.

During the preparation of SnF_2_ solutions with OMVs, some turbidity was observed. The samples were briefly centrifuged, revealing precipitation in the SnF_2_ and OMV tubes, but not in the OMV-alone tubes (Figure 6A). To characterize the interaction between OMVs and SnF_2_, we measured the turbidity (OD600) over time after mixing OMV and SnF_2_. The interaction was rapid, with turbidity increasing immediately upon the addition of SnF_2_ to the OMV solution (Figure 6B, *p* < 0.01). Supplementary Figure S3 shows that turbidity is dependent upon mutiple variables, including the concentrations of SnF_2_ and LPS. Free LPS was dramatically reduced when SnF_2_ increased from 0.25 to 10 mM in the reaction. Only a soluble fraction was analyzed in the mass spectrometer. Likely, the LPS was bound to SnF_2_ and precipitated.

### 3.4 Stannous binds LPS on surface of OMVs directly

**Figure 7:**
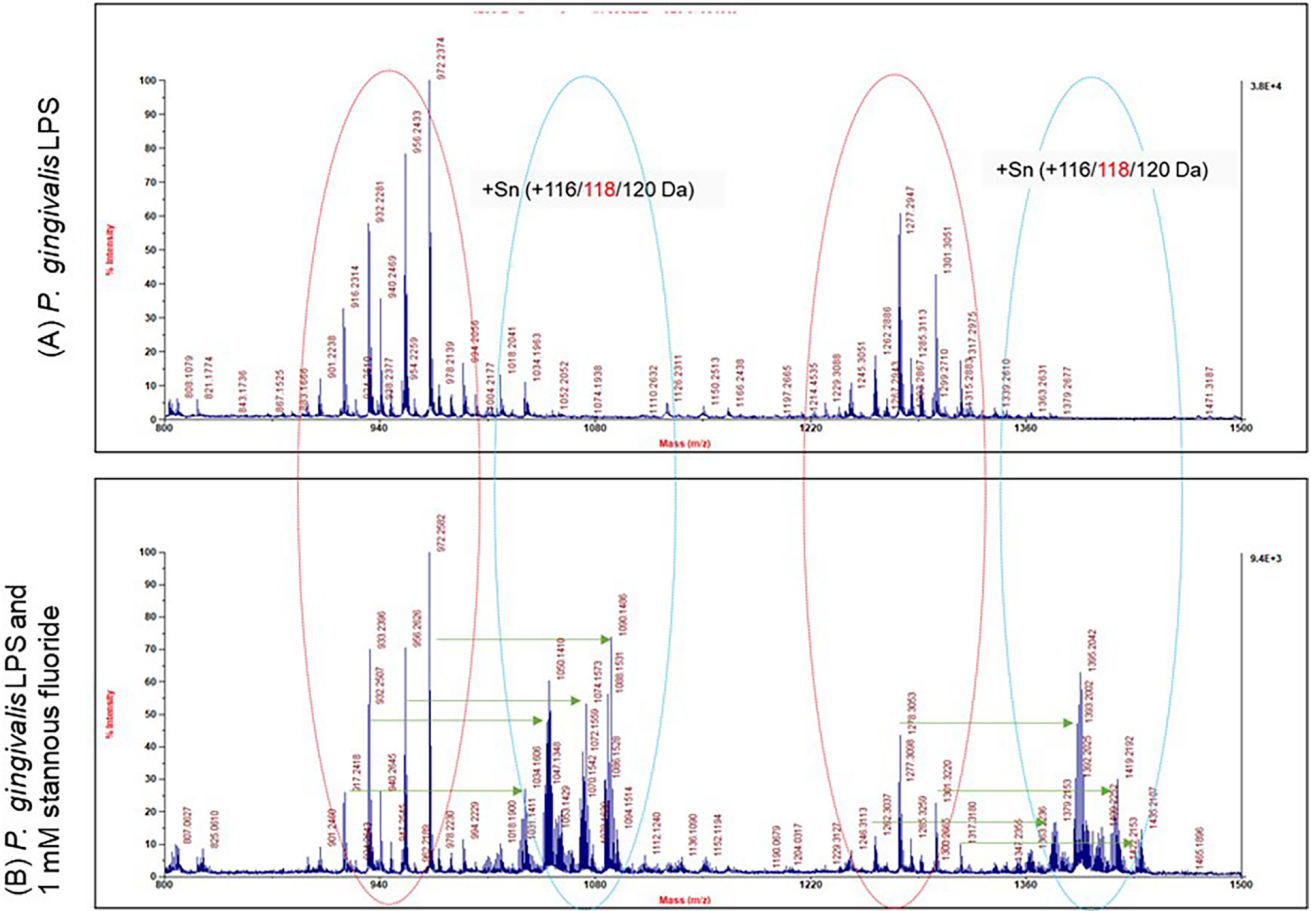
Mass spectrum analysis of stannous and LPS binding. *P. gingivalis* LPS is highly heterogeneous in size and structure. Each peak represents one molecule of LPS. There were two groups of LPS peaks in the LPS alone panel as indicated by the red circles. With SnF_2_, two more groups of peaks were generated as represented by the blue circles. The peaks in the blue circles were shifted by 116, 118 or 120 da to the right as indicated by the green arrows.

The precipitates formed with OMVs in the presence of SnF_2_ most likely result from interactions with LPS in the OMV membranes, given their high concentration in vesicles formed from *P. gingivalis*. We previously reported that *E. coli* LPS binds SnF_2_. In order to determine whether SnF_2_ bound directly to LPS on the OMV, we isolated LPS from a crude preparation and mixed it with SnF_2_. The direct reactivity of SnF_2_ with LPS from *P. gingivalis* was analyzed via mass spectrometry (Figure 7). Separation of *P. gingivalis* LPS following incubation with SnF_2_ produced signals associated with Sn atoms bound to the deposits. Tin has 10 stable isotopes, the highest number of any element. Three isotopes are most abundant (116 Sn, 118 Sn, 120 Sn), each about 9%. All three major tin isotopes were found to bind LPS, as the LPS peaks were shifted by 116, 118, and 120 Da (Figure 7). The molar ratio of the formed complexes appeared to be 1:1 (one stannous ion and one LPS molecule). Supplementary Figure S4 shows evaluations of both SnF_2_ and SnCl_2_ with ultrapure *E. coli* LPS. Results observed with *P. gingivalis* LPS and *E. Coli* LPS are similar in binding to SnF_2_.

### 3.5 Aggregation of *P. gingivalis* OMVs

**Figure 8:**
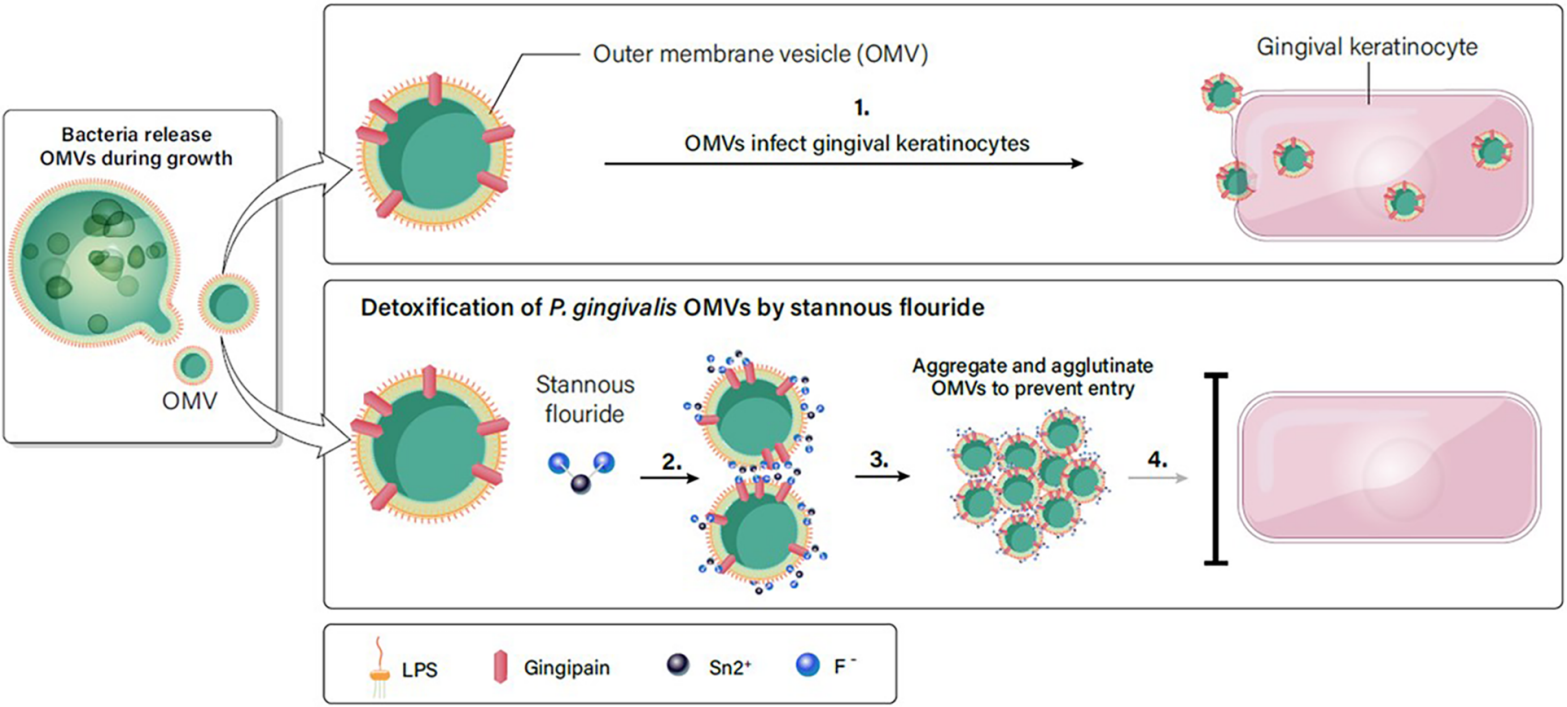
SnF_2_ interacts with OMVs to form aggregates. (1). OMVs are highly effective in entering gingival keratinocytes. (2). LPS on the surface of OMVs can interact with stannous, which is bivalent. Stannous can bind two OMV structures or bind one OMV and another stannous. (3). As more OMVs are linked together, they form a large aggregate. (4). Large aggregates of OMVs reduce the effective concentration of OMVs in the solution, and retard entry into gingival keratinocytes.

Based on the results in Figures 2–7, we propose a working model for how SnF_2_ inhibits the virulent effects of OMVs on gingival keratinocytes. OMVs are coated with LPS and other surface molecules. These molecules can bind to stannous ions. Stannous ions can also bind to each other, linking two OMVs together. As more OMVs aggregate, the effective concentration of OMVs is reduced. Additionally, larger particles may be less efficient at entering keratinocytes. Consequently, fewer OMVs gain entry into gingival keratinocytes (Figure 8).

## 4 Discussion

In this study we examined the reactivity of SnF_2_ and SnCl_2_ both in solution and in toothpaste extracts with OMVs produced by *P. gingivalis* cultures. In control experiments, OMVs from *P. gingivalis* were observed to infect suspensions of gingival keratinocytes and, following further incubation, the exposure of the gingival keratinocyte promoted the formation of ROS associated with promotion of an inflammatory response caused by OMV infection in the cells. ROS are generated in host tissues by the innate immune system in reaction to bacterial colonization, and OMVs provoke responses as illustrated herein. Invading pathogens and the surfaces of the OMVs can be recognized by pattern recognition receptors located on the surface of host cells. In test experiments, exposure of OMVs to SnF_2_ produced aggregated flocs which precipitated out of solution; these could be easily separated by centrifugation. The mixtures of OMVs with SnF_2_ solution or with SnF_2_ dentifrice extract demonstrated decreased infection and diminished development of ROS in gingival keratinocytes. Precipitation of OMVs by SnF_2_ could decrease their ability to create inflammation and likewise might diminish their migration to contribute to systemic sequalae.

The primary rationale for the efficacy of SnF_2_ in inhibiting OMVs of *P. gingivalis* presumably stems from the strong reactivity with endotoxin (LPS) components of the OMVs (Figure 8). OMV membranes are primarily comprised of LPS. In this study, the chemical binding of SnF_2_ to LPS was confirmed directly with TOF-MS. This complements our previous research where we observed that SnF_2_ was extremely effective in binding LPS and that treatment with the compound reduced the ability of endotoxin itself to potentiate inflammatory upregulation of toll receptors TLR2 and TLR4. In clinical studies, it was confirmed that SnF_2_ penetrated and was retained in the gingival sulcus (subgingivally) (42). Retained concentrations of SnF_2_ were in the same range as those shown in this study to produce deactivation of OMV toxicity. SnF_2_ reactivity with parent *P. gingivalis* bacteria produced aggregation in cell membranes of the bacterium and lysis as evidenced by TEM.

Gingival keratinocyte cells comprise the gingival epithelium, which functions as a physical barrier to prevent the invasion of periodontal-linked bacteria (43). *P. gingivalis* has been shown to infect keratinocytes, impairing cellular migration and proliferation as well as passing through the epithelial barrier into underlying tissues (44). TEM from invasion assays examining *P. gingivalis* cultured with gingival keratinocytes demonstrated *P. gingivalis* internalized within the keratinocytes (45). *In vitro* fluorescence imaging also demonstrated *P. gingivalis* localization within gingival keratinocytes (46). Bacterial infection appears to be in part mediated by proteolytic enzymes, as inhibition of proteases or deletion of gingipain genes significantly inhibits the ability of *P. gingivalis* to invade gingival keratinocytes (47, 48).

OMVs of *P. gingivalis* have gained prominence in the study of virulence factors associated with periodontal disease (26). Following colonization and growth of *P. gingivalis*, the outer membranes of the bacterium can form discrete OMVs by swelling with the outgrowths dislodging from the bacterium itself. These OMVs present with a protected membrane structure that researchers speculate assist in their evasion of degradation (26). Their numbers and size permit their transport while escaping deactivation by myriad protective factors of the host. A large number of virulence-related molecules are enriched in OMVs, including gingipains, LPS, Mfa5, PPAD, HmuY (49, 50). For example, gingipain concentrations on OMVs are 3 to 5 fold higher than on parent *P. gingivalis* bacteria (29). Gingipains themselves produce important pathogenicity, for example, in compromising barrier function of periodontal tissues (28). *P. gingivalis* OMVs enter cells such as gingival keratinocytes more effectively than the intact *P. gingivalis* bacterial cells (51). *P. gingivalis* OMVs swiftly enter host keratinocytes via an endocytosis pathway (52). Once OMVs infect gingival keratinocytes, cellular detachment is observed in a dose dependent manner (53). OMVs impair function of keratinocytes by degrading signaling molecules required for cell migration (54). OMVs activate pattern recognition receptors (PRRs) in gingival keratinocytes leading to cell activation, cytokine secretion and apoptosis (55). Specifically, OMV infection of keratinocytes stimulates increased expression of pro-inflammatory mediators including COX-2, IL-6, IL-8, MMP-1 and MMP-3 (56).

**Figure 9:**
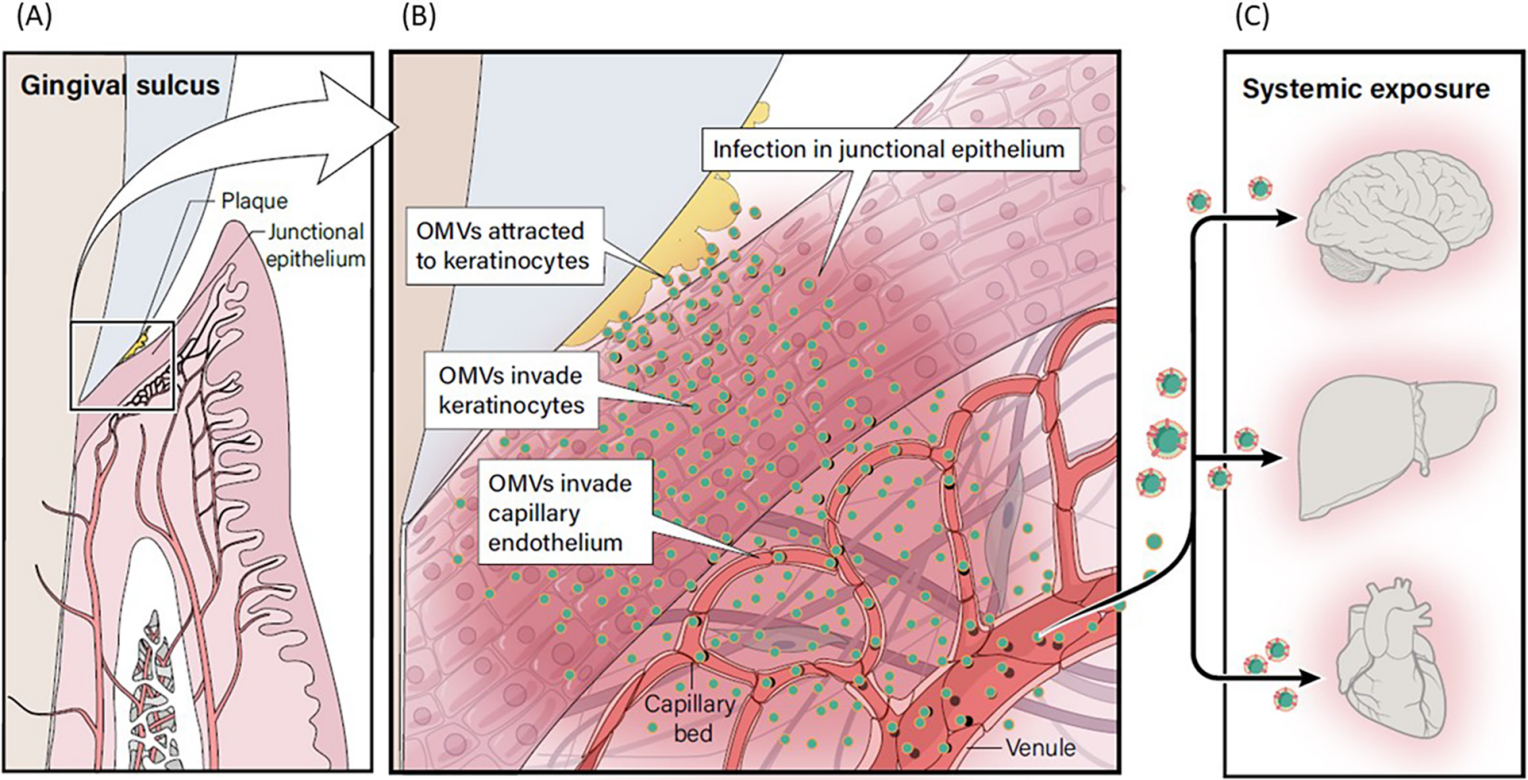
Proposed model for relationship between OMV infection and systemic exposure. **(A)** *P. gingivalis* within plaque in the gingival sulcus produces OMVs. **(B)** OMVs adhere to gingival keratinocytes, leading to invasion of keratinocytes. **(C)** OMVs can migrate through the disrupted epithelial barrier and into the underlying capillary beds creating a potential pathway to introduce OMVs into the circulatory system where they could be carried to distant tissues and organs, including the brain, liver and heart.

Contemporary research suggests that *P. gingivalis* OMVs may potentially influence a variety of systemic diseases through their transport to various distant target organs (Figure 9). *P. gingivalis* OMVs can migrate through the epithelial barrier and into the underlying capillary beds introducing OMVs into the circulatory system, carrying OMVs to distant tissues and organs (57). They promote increased vascular permeability and enhance vascular edema through proteolytic damage to endothelial connexins (58). *P. gingivalis* OMVs also have powerful platelet aggregation activity, which is an important mechanism in the formation of atherosclerotic plaque formation (59, 60), and they carry gingipain to the liver and alter glucose metabolism in the liver, promoting the development of Diabetes Mellitus (61). *P. gingivalis* OMV derived LPS can activate glial cells, induce brain inflammation, and correlate with the expression of Alzheimer's Disease marker proteins and neorfibrillary tangles (62). A proposed model for the potential relationship between OMV infection of gingival keratinocytes and subsequent infection of OMVs into underlying connective tissue and capillary beds with increasing systemic exposure is shown in Figure 9. Further research is needed to evaluate the role that *P. gingivalis* OMVs play in the initiation and progression of periodontal diseases and their relevance to putative oral disease—systemic disease linkage. Definitive evidence will require randomized, well controlled clinical research targeting the role of OMVs in disease pathogenesis. Deactivation of OMVs could present an important target for chemotherapeutics in prevention of periodontal diseases and limiting systemic exposure to OMVs of oral origin.

## 5 Conclusion

This study showed that OMVs from *P. gingivalis* are an infective and dose-dependent virulence factor. They are shown to invade host keratinocyte cells inducing the production of ROS by the keratinocytes. OMVs contain large concentrations of LPS and, in fact, endotoxins isolated from culture media are likely derived from OMVs rather than parent bacteria. Stannous and 0.454% SnF_2_ toothpaste can bind both OMVs and LPS, inhibiting the infection of keratinocytes and induction of ROS production. Reactivity of OMV LPS with stannous is likely to aggregate OMVs together, thereby precipitating out in the solution. Large aggregates of OMVs reduce infectivity.

## Data Availability

The raw data supporting the conclusions of this article will be made available by the authors, without undue reservation.
