# Supplementary material for: Stannous fluoride protects gingival keratinocytes against infection and oxidative stress by Porphyromonas gingivalis outer membrane vesicles
Source: Front Dent Med. 2024 Nov 19;5:1492369. doi: 10.3389/fdmed.2024.1492369 (PMC11797948; doi:10.3389/fdmed.2024.1492369)

Supplementary figures

**Supplementary Figure 1. Effects of *P. gingivalis* OMV infection and stannous treatment on cell confluence.** A. Gingival keratinocytes were subjected to a range of *P. gingivalis* outer membrane vesicle (OMV) concentrations. Over a 42-hour observation period, cell confluence was measured using phase contrast microscopy. The statistical analysis indicated no significant variation in cell confluence among the different OMV doses, which were equivalent to protein concentrations. B. Gingival keratinocytes received treatments with *P. gingivalis* OMVs, stannous fluoride (SnF_2_), or stannous chloride (SnCl_2_). Cell confluence was monitored using phase contrast microscopy over 42 hours. The results showed no significant differences in cell confluence across the various doses, mirroring the OMV-only treatment. The findings, expressed as mean ± standard error (SE) from three separate experiments, were visualized using the ggpubr package in RStudio. One-way ANOVA and t-tests were employed to assess statistical significance, comparing each treatment to the OMV-only control group (0 μM stannous).


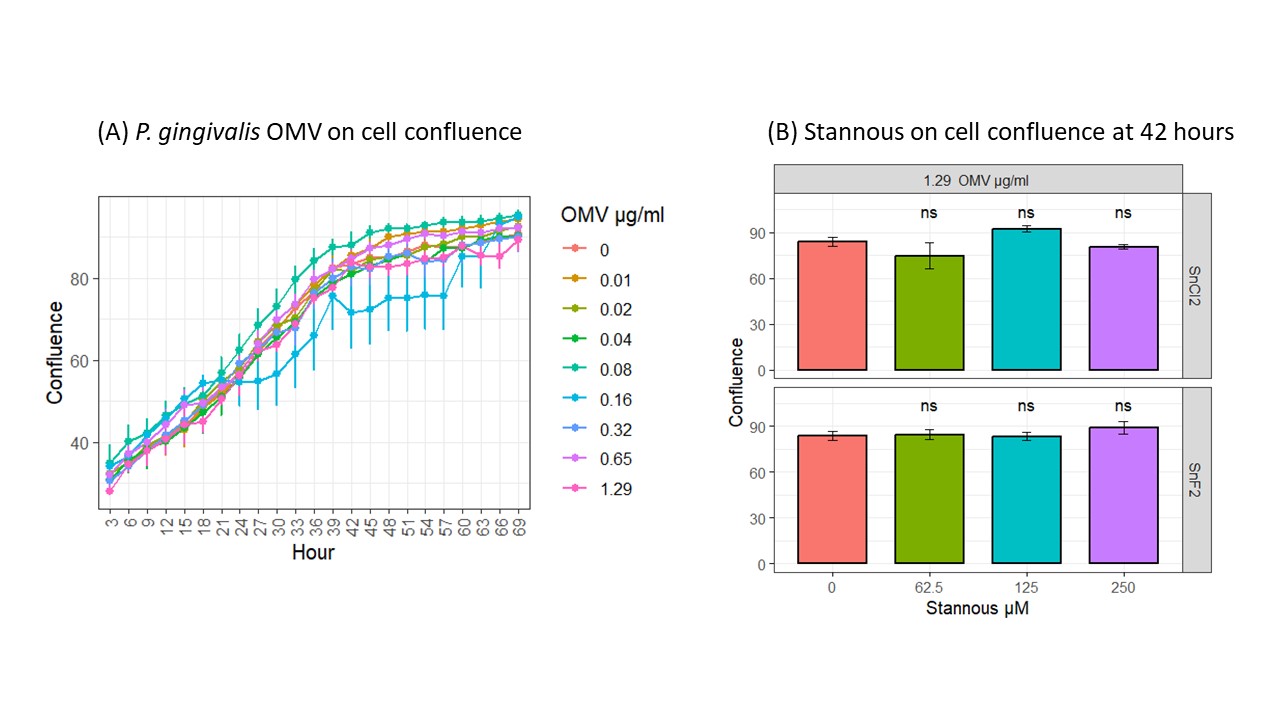


**Supplementary Figure 2. Effects of SnF_2_, SnCl_2_, and stannous-containing toothpastes on cell confluence.** Gingival keratinocytes were treated with 0.65 µg/ml *P. gingivalis* outer membrane vesicles (OMVs), along with SnF_2_ (A), SnCl_2_ (B), and SnF_2_-containing toothpastes (C). Cell confluence was quantified using phase contrast microscopy. The results, depicted as mean ± standard error (SE), were derived from four independent experiments.


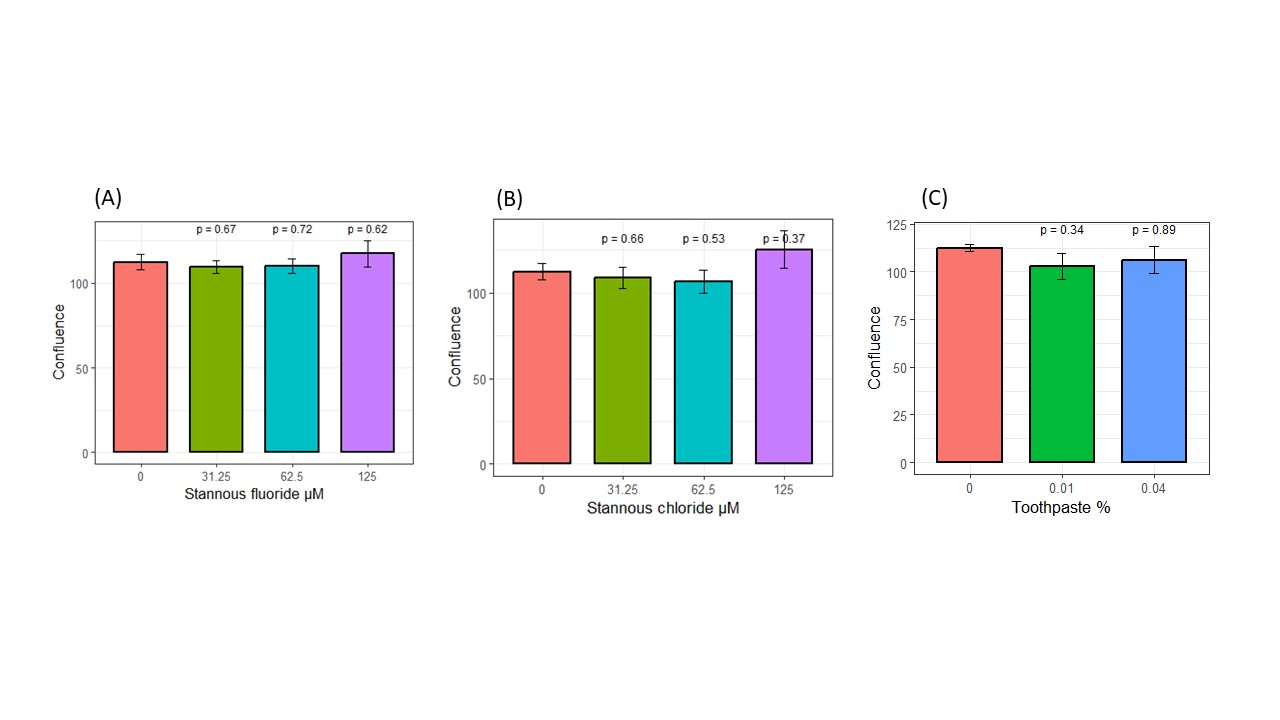


**Supplementary Figure 3. SnF_2_ at different concentrations binds *P. pallens* LPS.** LPS of *P. pallens* were prepared using the same procedures described in the Materials and Methods section. *P. pallens* ATCC 700821 was cultured using procedures described in Xie S et al. SnF_2_ forms aggregates between outer and inner membranes leading to membrane rupture of *Porphyromonas gingivalis* and *Prevotella pallens*. *Front Oral Health*. (2024) 5:1427008. doi:10.3389/froh.2024.1427008. To determine the optimal dose of SnF_2_ used in the LPS binding assay, we ran doses of SnF_2_ with *P. pallens* LPS. *P. pallens* LPS was isolated from bacterial cells. *P. pallens* LPS (120, 000 endotoxin units) was mixed with SnF_2_ at 0.2, 1 and 5 mM. The tubes were set for 10 min at room temperature, and then were centrifuged at 5000 RPM for 5 min to pellet the precipitates using a table microfuge. The supernatants were investigated via MALDI TOF. Two groups of LPS were detected in the mass spectrum between 800 and 1500. SnF_2_ at 2 mM added two groups of peaks that trailed the LPS peaks by 116, 118 and 120 daltons, indicating binding one atom of tin to each molecule of LPS moiety (tin-LPS). It is worth noting the LPS peaks and tin-LPS peaks became smaller if SnF_2_ increased to 5 and 10 mM. The results suggested high concentrations of SnF_2_ precipitated LPS out of solution.

**
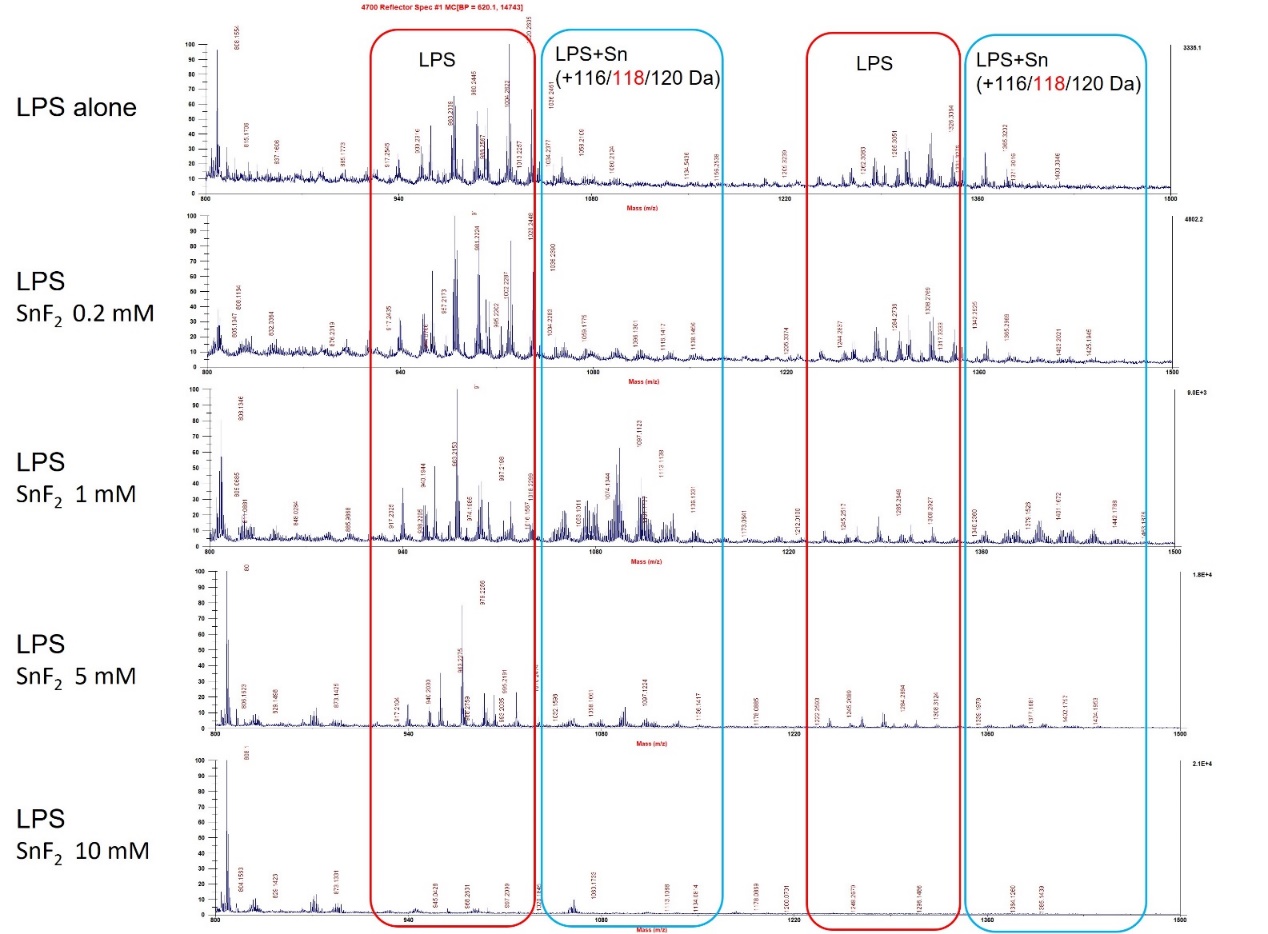
**

**Supplementary Figure 4. SnF_2_ and SnCl_2_ bind ultrapure *E. coli* LPS**. Ultrapure *E. coli* LPS were purchased from Invivogen (San Diego, CA). SnF_2_ and SnCl_2_ binding to LPS was performed as decribed in the Materials and Methods section.


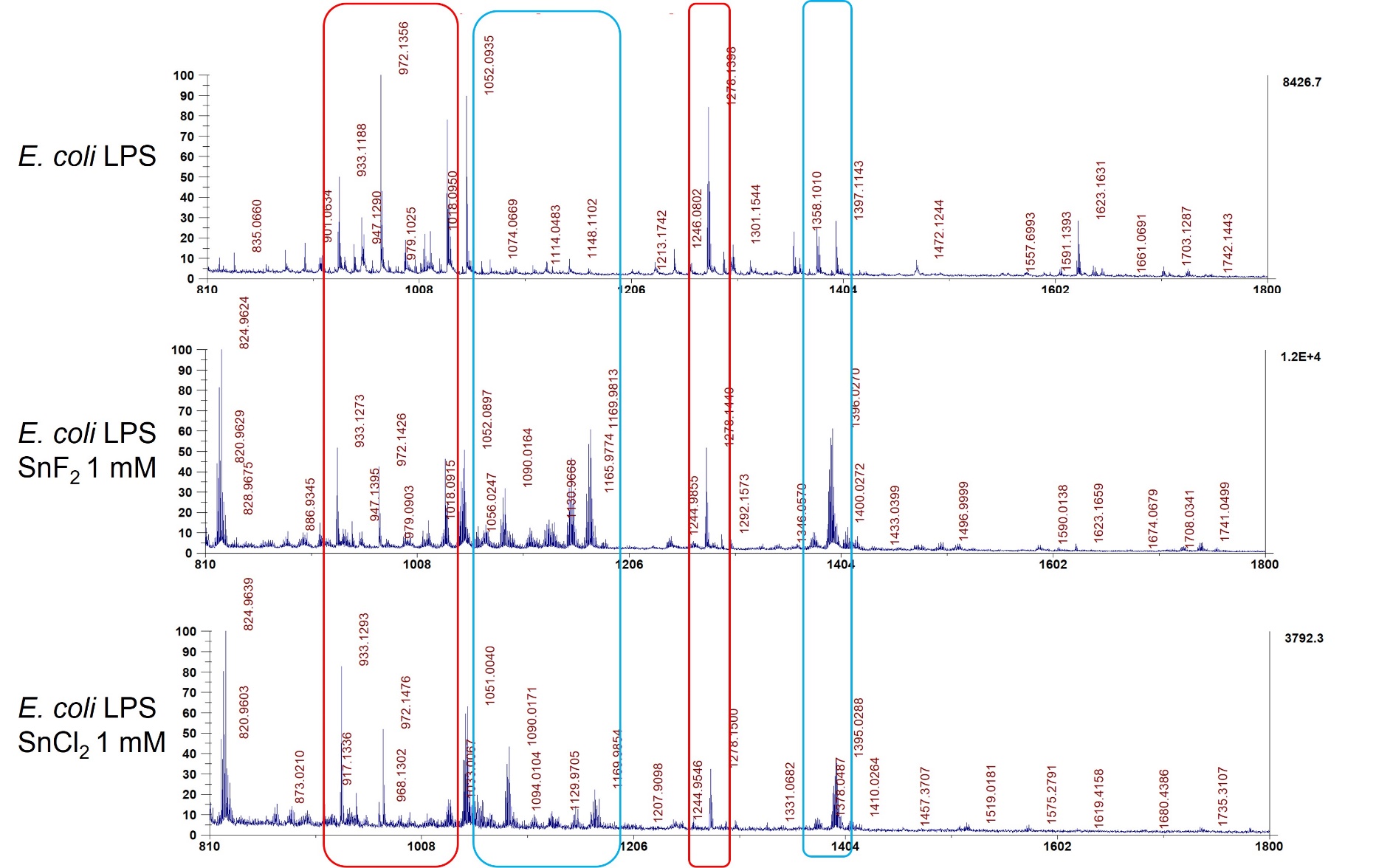

Supplement: Supplementary file 1 [file Datasheet1.docx]
